# The role of undergraduate medical students training in respect for patient confidentiality

**DOI:** 10.1186/s12909-021-02689-6

**Published:** 2021-05-12

**Authors:** Cristina M Beltran-Aroca, Rafael Ruiz-Montero, Fernando Labella, Eloy Girela-López

**Affiliations:** 1 Sección de Medicina Legal y Forense, Facultad de Medicina y Enfermería, Universidad de Córdoba, 14004 Córdoba, Spain; 2 Instituto Maimónides de Investigación Biomédica de Córdoba (IMIBIC), Hospital Universitario Reina Sofía, Universidad de Córdoba, Avda. Menéndez Pidal s/n, 14004 Córdoba, Spain; 3 Sección de Oftalmología, Facultad de Medicina y Enfermería, Universidad de Córdoba, 14004 Córdoba, Spain

**Keywords:** Professionalism, Undergraduate, Medical students, Confidentiality, Ethical issues

## Abstract

**Background:**

Encouraging professional integrity is vital for providing a standard of excellence in quality medical care and education and in promoting a culture of respect and responsibility. The primary objective of this work consisted of studying the relationship of medical students to the right to patient privacy in Spain, specifically by analysing the conditions for accessing patient clinical histories (CHs).

**Methods:**

A cross-sectional study was conducted based on a questionnaire sent by e-mail to final-year students at 41 Spanish universities. It had 14 multiple choice and closed questions framed in 3 large blocks. The first question addressed basic general knowledge issues on the right to privacy and the obligation for confidentiality. The two remaining blocks were made up of questions directed towards evaluating the frequency with which certain requirements and action steps related to students attending patients were performed and regarding the guarantees associated with accessing and handling patient CHs both on paper and in the Electronic Medical Record.

**Results:**

A total of 245 valid replies were considered. A total of 67.8 % of participants were women, with an average age of 24.05 ± 3.49 years. Up to 90.6 % were aware that confidentiality affected the data in CHs, although 43.3 % possessed non-anonymized photocopies of patient clinical reports outside the healthcare context, and only 49.8 % of the students were always adequately identified. A total of 59.2 % accessed patient CHs on some occasions by using passwords belonging to healthcare professionals, 77.2 % of them did not have the patients’ express consent, and 71.9 % accessed a CH that was not anonymised.

**Conclusions:**

The role of healthcare institutions and universities is considered to be fundamental in implementing educational measures regarding the risks and ethical and legal problems arising from the use of CHs among professionals and students. A thorough study of medical ethics is needed through the analysis of clinical cases and direct exposure to situations in which the patient’s confidentiality is questioned.

**Supplementary Information:**

The online version contains supplementary material available at 10.1186/s12909-021-02689-6.

## Background

The right to privacy is inherent to human dignity. Together with freedom and private life, it signifies respect for the individual’s autonomy [1]. Without an expectation of privacy, patients might not disclose important information or may avoid seeking care, fearing a loss of employment or stigmatization [2]. The rights to privacy and the protection of health data deserve particular respect in the healthcare environment [3]. This duty primarily falls on the healthcare professional in charge of attending the patient, but we cannot obviate the fundamental role of universities and healthcare institutions in training future professionals. It is crucial to promote professional integrity with the aim of providing a standard of excellence in care quality and at a medical education level, of fostering a culture of respect and responsibility [4]. Various publications have noted the importance conferred by medical students to the patient’s right to privacy [5–7]. In Spain, a considerable number of students come into contact with patients and their respective personal data through clinical practice sessions in different healthcare centres. This characteristic inspired the publication of the *Patient Privacy Protocol* in 2017 [8], in which promoting respect for patient confidentiality stands out, and it affects all health sciences students [9, 10]. The obligation of medical students is to respect the confidentiality of the information contained in the clinical history (CH) of patients strictly, regardless of its use. The exemplary duty of those responsible for clinical teaching should be added when considering that any medical act enshrines an important ethical value at all times [11]. They should supply the students with all the opportunities necessary for learning professionalism during clinical practice classes, taking maximum advantage of the value of the hidden curriculum [12].

The primary objective of this work consisted of studying the relationship of medical students to the right to patient privacy. Specifically, specific assumptions were analysed with respect to the conditions of access to information in CHs by means of a questionnaire that we prepared.

## Methods

A cross-sectional, descriptive, and observational study was conducted based on a questionnaire sent to students who were doing their clinical practice sessions in the final year (6th ) of medicine studies at 41 Spanish universities. The reference population comprised all 6th -year medicine students in Spain during the 2019/20 academic year, who began their studies in 2014/2015. In 2014/15, 7,127 people enrolled in the degree in medicine in Spain, according to data from the Ministry of Science, Innovation and Universities [13]. Through this source, the reference population was obtained by the university. A sample by quotas (university) was conducted to ensure the correct distribution of the sample obtained in the survey. A sample size composed of the sum of the quotas was calculated. In a random sample, 258 individuals were sufficient to estimate with a 95 % confidence level, a population percentage of approximately 50 %.

A first draft of the questionnaire was submitted to a panel of experts that was composed of 4 doctors. After their suggestions for revision were received, the questionnaire was revised and modified. Next, the questionnaire was evaluated by means of a pilot survey from 20 students of medicine at the University of Córdoba, resulting in another series of changes that were related to some of the practical aspects of its administration. Responses from a pilot study were excluded from the final statistical analysis. To recruit participants, a final questionnaire was sent to students by e-mail. An anonymous answer stored in computerized form was generated automatically, to which only the experts had access. The period for turning in the sample was from November 2019 to March 2020.

The questionnaire included a series of sociodemographic characteristics: sex, age, and the university at which the participant performed their practical work during the 2019/20 academic year. The questionnaire (see Additional file 1) had 14 multiple choice and closed questions. Some of them were directly related to the *Patient Privacy Protocol* with regard to student respect for the right to the privacy of the patient, and they were framed in 3 blocks. The first one was made up of the first two questions (Q-no. 1–2) and addressed general knowledge issues on the right to privacy and the obligation of confidentiality. The two remaining blocks were made up of questions directed towards evaluating the frequency (always, often, sometimes, seldom, or never) with which specific action requirements related to the students were performed when attending a patient (Q-no. 3–8) and regarding the guarantees in accessing and managing their CH both on paper and in the Electronic Medical Record (EMR) (Q-no. 9–14). It was presented in four languages: Spanish, Catalan, Galician and Basque.

The questionnaire and methodology for this study were performed in accordance with the Declaration of Helsinki and were approved by the *Human Research Ethics Committee of the University of Córdoba* (Spain) (Ref. No. CEIH-20-21). Students were told that their participation in the study was voluntary and that there was a guarantee of confidentiality and anonymity. Additionally, our study adheres to STROBE guidelines (see Additional file 2) for reporting observational research.

A statistical analysis was performed with PASW Statistics 25 software (IBM SPSS®). In addition to the descriptive analysis, a comparison of proportions was made between the different groups by Chi-squared (χ2) tests for contingency tables. Lastly, a binomial logistic regression or an ordered logistic regression was conducted, as appropriate, according to sex (crude) and sex + age (adjusted odds ratios). The values considered to be statistically significant were those with a level of confidence of over 95 % (*p* < 0.05). A quality control was performed to ensure that the resulting sample had a similar distribution of frequencies per age group and sex compared to that of the reference population.

## Results

Of the 7,127 students entering the degree in medicine during the 2014/15 academic year, 6.7 % (*N* = 474) fully answered the questionnaire in 2019/20 (incomplete questionnaires were not evaluated). A random selection was made to study the number of respondents representing each university according to the number of places offered, reaching a total of 245 valid responses. A total of 90.2 % (*n* = 37) of the 41 universities satisfied the optimal amount required for the questionnaires, but we did not receive any response from 7.3 % of them (*n* = 3) (Table 1). The general data in the sample were representative of the population that we started from; 67.8 % (*n* = 166) of the participants were women, and the average age was 24.05 ± 3.49 years old (range 19–56).

**Table 1:** Questionnaires (Q) requested and ultimately received in accordance with the number of places offered by each university

	Places offered^a^	Q. Requested		Q. Received	
		n	%	n	%
Universidad Alfonso X el Sabio	120	4	1.6	4	1.6
Universidad Autónoma de Madrid	275	10	3.9	10	3.9
Universidad Católica de Murcia	90	3	1.2	3	1.2
Universidad Católica de Valencia	119	4	1.6	4	1.6
Universidad CEU Cardenal Herrera	120	4	1.6	4	1.6
Universidad CEU San Pablo	160	6	2.3	6	2.3
Universidad Complutense de Madrid	320	12	4.7	12	4.7
Universidad de Alcalá	373	14	5.4	14	5.4
Universidad de Cádiz	155	6	2.3	6	2.3
Universidad de Cantabria	120	4	1.6	4	1.6
Universidad de Castilla La Mancha (Albacete)	115	4	1.6	4	1.6
Universidad de Castilla La Mancha (Ciudad Real)	60	2	0.8	2	0.8
Universidad de Córdoba	120	4	1.6	4	1.6
Universidad de Extremadura	120	4	1.6	4	1.6
Universidad de Granada	253	9	3.5	9	3.5
Universidad de la Laguna	130	5	1.9	5	2.0
Universidad de las Palmas de Gran Canaria	135	5	1.9	5	2.0
Universidad de Málaga	170	6	2.3	6	2.3
Universidad de Murcia	200	7	2.7	7	2.7
Universidad de Navarra	210	8	3.1	8	3.1
Universidad de Oviedo	150	5	1.9	0	0
Universidad de Salamanca	203	7	2.7	7	2.7
Universidade de Santiago de Compostela	350	13	5.0	13	5.0
Universidad de Sevilla	320	12	4.7	12	4.7
Universidad de Valladolid	185	7	2.7	7	2.7
Universidad de Zaragoza	230	8	3.1	8	3.1
Universidad del País Vasco	270	10	3.9	10	3.9
Universidad Europea de Madrid	200	7	2.7	7	2.7
Universidad Francisco de Vitoria	120	4	1.6	4	1.6
Universidad Internacional de Catalunya	90	3	1.2	2	0.8
Universidad Miguel Hernández	130	5	1.9	5	1.9
Universidad Rey Juan Carlos	150	5	1.9	5	1.9
Universitat Autònoma de Barcelona	320	12	4.7	12	4.7
Universitat de Barcelona (C. Bellvitge)	140	5	1.9	5	1.9
Universitat de Barcelona (C. Clínic)	119	4	1.6	4	1.6
Universitat de Girona	80	3	1.2	3	1.2
Universitat de Lleida	120	5	1.9	0	0
Universitat de València	320	12	4.7	12	4.7
Universitat Jaume I	80	3	1.2	3	1.2
Universitat Pompeu Fabra	60	2	0.8	0	0
Universitat Rovira i Virgili	125	5	1.9	5	1.9
Total	7127	258	100	245	100

^a^Places offered by each University in the academic year 2014/15

### Learning professional values

The first question refers to the right to privacy. Up to 88.2 % answered that they were familiar with the confidential nature of health data as well as the idea that information that patients had revealed and confided to them was private (73.9 %). A total of 61.2 % responded correctly, i.e., indicating all the options, but no statistical significance was observed in terms of the participant’s sex (*p* = 0.889).

When asked about the obligation of confidentiality in Q-no. 2, 76.7 % correctly marked all the options, with statistical significance being observed in favour of men (*p* = 0.042) (Table 2). The majority percentage came from the option contained in the CH (90.6 %), followed by maintaining that obligation even after the death of the patient (89.4 %).

**Table 2:** Crude and adjusted odds ratios (and 95 % confidence intervals) from logistic regression analyses for identifying associations between question, sex, and age

	Crude odds ratio		Adjusted odds ratio			
	Sex (ref = Female)		Sex + Age (ref = Female)			
	Sex	p	Sex	p	Age	p
Q1. Right to privacy	1.05 (0.60–1.83)	0.859	1.04 (0.60–1.82)	0.885	1.01 (0.94–1.10)	0.727
Q2. Obligation of confidentiality	2.08 (1.05–4.35)	0.042^*^	2.16 (1.09–4.56)	0.034^*^	0.96 (0.88–1.04)	0.276
Q3. Commitment to confidentiality	0.97 (0.48–2.01)	0.931	0.93 (0.46–1.94)	0.839	1.10 (0.97–1.36)	0.286
Q4. Student knows the tutor	1.24 (0.75–2.06)	0.400	1.24 (0.75–2.06)	0.402	1.00 (0.93–1.08)	0.998
Q5. Wearing an identification tag	0.88 (0.53–1.46)	0.615	0.84 (0.51–1.40)	0.506	1.08 (1.00-1.21)	0.110
Q6. Negative consequences	1.81 (0.49–6.40)	0.353	1.94 (0.52–6.94)	0.303	0.84 (0.53–1.14)	0.421
Q7. Patient knows you are a student	1.21 (0.73–2.01)	0.471	1.19 (0.72–1.99)	0.496	1.04 (0.97–1.11)	0.303
Q8. More than 4 students	0.96 (0.59–1.57)	0.868	0.95 (0.58–1.56)	0.839	1.05 (0.95–1.16)	0.361
Q9. Access to patient EMR	1.78 (1.02–3.16)	0.045^*^	1.75 (1.00-3.12)	0.052	1.03 (0.95–1.13)	0.519
Q10. Consent of the patient to access	0.80 (0.34–1.79)	0.598	0.72 (0.30–1.64)	0.445	1.11 (1.00-1.27)	0.068
Q11. Access to patients dissociated CH	0.89 (0.54–1.47)	0.657	0.88 (0.53–1.45)	0.624	1.03 (0.95–1.12)	0.624
Q12. Non-anonymized photocopies	1.15 (0.67–1.97)	0.616	1.15 (0.67–1.98)	0.609	1.00 (0.92–1.07)	0.899
Q13. Consent of the patient to have photocopies	1.17 (0.40–3.23)	0.770	0.79 (2.28–2.39)	0.686	1.33 (1.07–1.73)	0.024
Q14.Anonymisation for FYP	1.50 (0.82–2.85)	0.198	1.49(0.81–2.84)	0.207	1.01 (0.93–1.12)	0.817

^*^p value: *p* < 0.05; Ref: reference group

### Requirements of action to be followed when attending patients

For Q-no. 3, 78.8 % affirmed having signed a commitment of confidentiality during their practical exercises.

When asked whether they knew the person in charge of supervising their practical classes in Q-no. 4, and 75.9 % answered positively as a fact that occurred regularly.

**Fig. 1:**
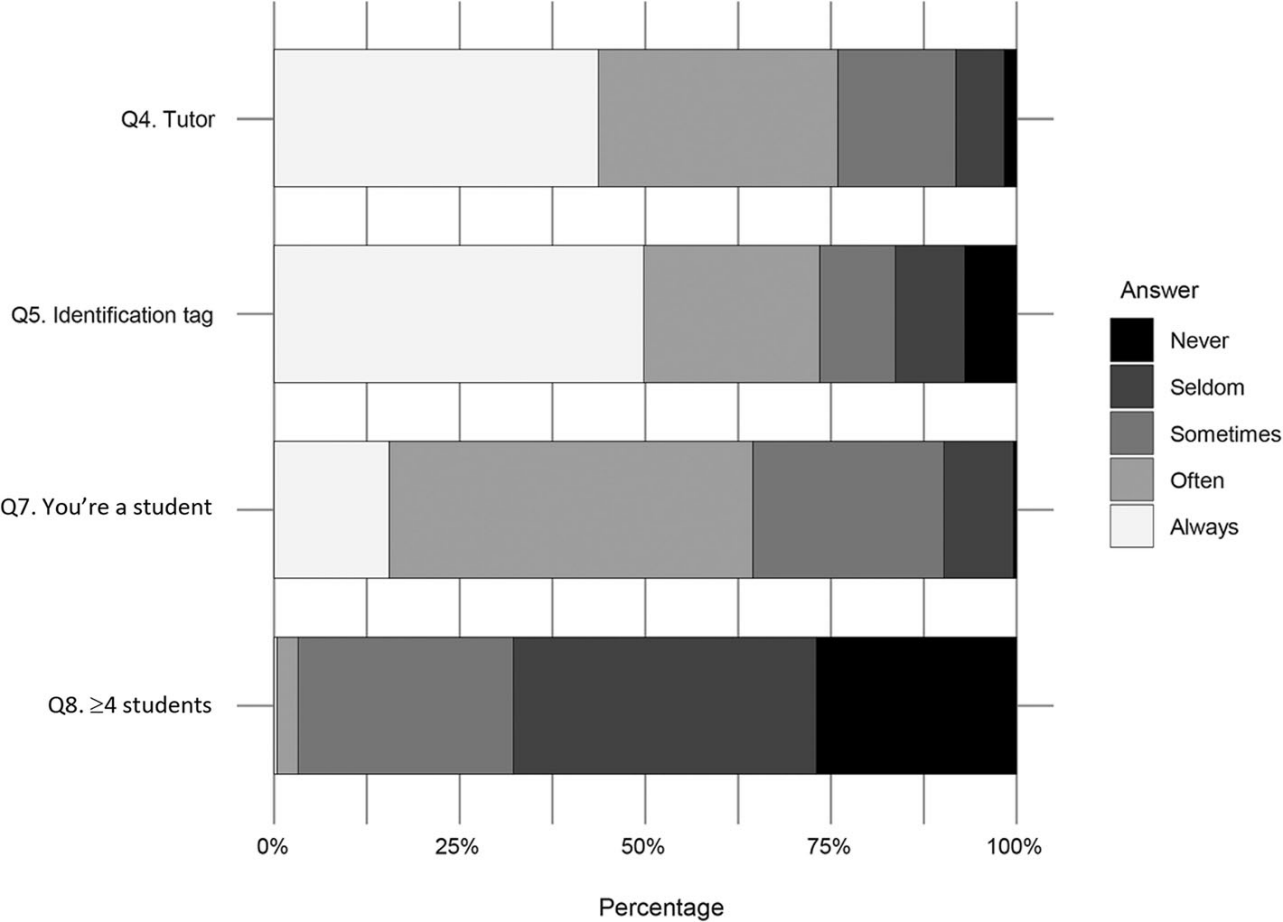
Frequency of the requirements demanded with respect to students when attending patients. Q: question

For Q-no. 5, 73.5 % claimed to wear an identification tag usually during their practice classes, and no statistical significance was observed in relation to the sex of the subject (*p* = 0.702) (Fig. 1). However, in Q-no. 6, most of those who admitted not always wearing it (*n* = 123) indicated that this action did not trigger any negative consequence for them from their tutor (90.2 %).

**Fig. 2:**
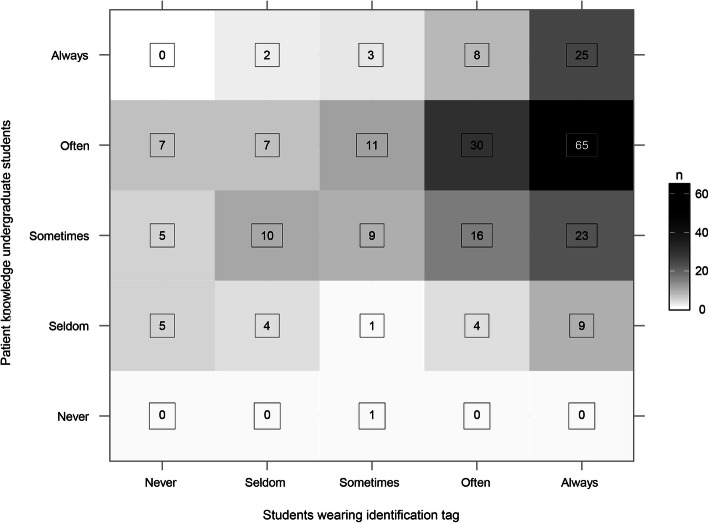
Frequency with which patients identify the students from their identification tag (*p = 0.002)

In Q-no. 7, 49 % reported that patients could often identify them as a student to an adequate extent, and 25.7 % only sometimes (Fig. 1). Among those students who usually wore identification, 71.1 % were generally identified by patients (*p* = 0.002) (Fig. 2).

When asked whether there were more than 4 students attending a patient at the same time in Q-no. 8, they answered that it was rare (67.7 %) (Fig. 1).

### Guarantees in accessing clinical history

For Q-no. 9, 59.2 % (*n* = 145) stated that they had accessed the EMR of patients using an authentication mechanism supplied by a healthcare professional. This response was more frequent among men (*p* = 0.045) (Table 2). Only 16.6 % of them had obtained the patient´s consent (Q-no. 10).

In Q-no. 11, 71.9 % of the students referred to accessing patients’ CHs with personal data dissociated from clinical data occasionally. No statistical significance (*p* = 0.945) was found with respect to those who had accessed the CHs.

For Q-no. 12, 43.3 % (*n* = 106) possessed non-anonymized photocopies of the patient’s clinical record outside the health centre. No significant differences were observed between those who possessed photocopies and those who answered questions 1 (*p* = 0.188) and 2 (*p* = 0.649) correctly. Only 10.4 % of those who claimed to have this type of photocopies claimed to have the patients’ express consent (Q-no. 13).

**Fig. 3:**
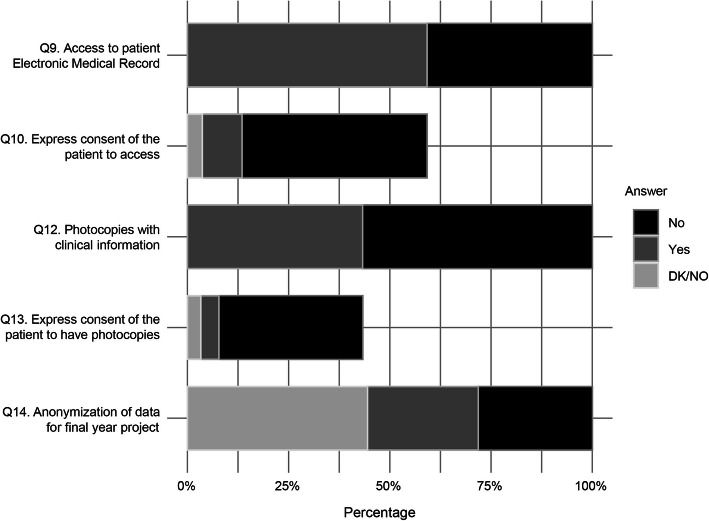
Access to and management of clinical documentation by the student. Q: question

For students who answered Q-no. 14 because their Final Year Project (FYP) involved the use of patient clinical databases for research (*n* = 136), 49.3 % indicated that the given data were anonymized (Fig. 3).

All the previously described results were adjusted for sex and age. The values are shown in Table 2.

## Discussion

### Learning professional values

The *World Federation for Medical Education* emphasizes the need to balance the academic capacities and the behaviours of medical students. The aim is to assist them in undertaking life-long learning and demonstrating their professionalism in the different roles of a doctor [14]. Professionalism includes respect for the confidentiality of patients, which is one of the basic skills that they have to develop and maintain both as undergraduate medical students and as doctors during their professional careers [15].

A total of 88.6 % of the respondents were aware that the obligation of confidentiality affected the most intimate physical and mental health data, also when considering the contents of CHs (90.6 %), such as the results of medical examinations, complementary tests, or genetic data. The constant development of genetic testing technologies has promoted the objective that medical students know about the special protections for these data to avoid any type of discrimination in the family, social or work environment [16]. Notably, it also includes any information assigned to a physical person for health purposes that identifies them univocally [17]. The question about this issue had a majority response, although it was not frequently selected by the students (85.7 %). Recently, some deficiencies have been described in relation to knowledge about the obligation of confidentiality [7, 18, 19]. Other authors mention final-year students who show little respect for patient confidentiality compared to other obligations, which is more marked in male students [20]. However, in this study, the men responded significantly more correctly to the concept of confidentiality. In recent decades, the teaching of medical ethics has been implemented as a priority in the undergraduate curriculum of medical schools worldwide. Social changes and further developments in science and technology have contributed decisively to this change [21]. Moreover, it has become evident that medical students lose their ability to recognize ethical dilemmas and to address these situations with empathy and moral reasoning during medical education [22]. The need to reinforce competency-based education [23] in study plans has been proposed as well as implementing measures oriented towards developing the study of medical ethics. This subject presents some differentiated characteristics, so it would be necessary to go deeper into the analysis of professional conduct guides [18]. It is not suggested that medical schools replace more traditional bioethical theoretical teaching with the teaching of everyday ethics but rather to consider it a necessary supplement [24]. Thus, theoretical training is recommended, which is based on the analysis of clinical cases in which problems related to confidentiality arising in clinical practice are posed as well as direct exposure to situations in which the patient’s privacy is questioned [25, 26].

### Requirements of action to be followed when attending patients

Medical students confirm their obligation to respect the human dignity, freedom of choice and privacy of the patient [17] by signing a commitment to confidentiality at the beginning of the practice exercise period in their healthcare institution. The schools of medicine should inform the healthcare institution about the students who are going to do practice exercises. Most of the participants did so, although a non-negligible percentage admitted that they had not (17.1 %).

According to the principle of distributive justice, the benefits and burdens of health care, research, and education should fall fairly on all members of society. Society expects competent and well-trained health professionals. The direct intervention of students in patient health is a key element, because current patients are likely to benefit from the previous experiences of these practitioners [27]. Thus, it would be vital for both the patients and healthcare professionals to be aware of the presence of persons in training during patient attendance and that the institution takes on the responsibility of giving the student a card/tag permitting their identification [8]. Patients confer great importance to knowing who is participating in the medical process [28], and this study shows that only approximately half of the students were always suitably identified, and a large number of them who did not wear the tag (90.2 %) did not refer to any negative reactions from their tutor. The presence of students may also be a conditioning factor, which raises different opinions. Some professionals contend that patients cannot refuse their intervention in an educational institution such as a university teaching hospital, whereas others believe that there is a direct presumption of patient consent if the latter does not actively oppose it. The *Protocol* demands that express consent should be obtained authorizing their presence during medical attendance and that their number should be limited in attending the same patient [8]. Most patients usually accept the participation of students [29], although in certain specialist treatments, the patient’s response could be conditioned by their sex [30]. This study did not permit us to make that inference, but 64.5 % declared that the patients usually expressly knew that they were students, and this knowledge was significantly related to wearing a tag.

To ensure the fulfilment of all the described requirements, the healthcare institution itself will designate a tutor who will be the person of reference whom the student should address [8]. Since honesty has been found to be essential in the doctor-patient relationship, tutors should disclose and honestly explain the role of anyone who is present during a medical encounter, indicating those who are present for education as a medical professional [27]. Despite the circumstances in the healthcare system not always being favourable, the figure of the tutor is considered to be highly relevant [30], not only as a supervisor but also for feedback and assessment work with the student when acquiring practical skills in a safe and thoughtful atmosphere [31, 32]. A total of 75.9 % of the participants usually knew who the tutor in charge was during the practice exercises, which was especially positive.

### Guarantees in accessing clinical history

The CH is an instrument that is fundamentally aimed at guaranteeing adequate attendance of the patient. Digital support permits better legibility and accessibility and a more efficient and accurate organization of the data with respect to those on paper [33]. The *Protocol* expressly prohibits EMR access to students [8]. The reasons could be based on the fact that in CHs on paper, it is not possible to look for the records of different patients at the same time or for several medical attendance episodes in the same individual, or easily duplicate or edit the data [34]. These results contrasted significantly with the legal precepts, since over half of the participants indicated that they accessed the CH of patients on some occasions (59.2 %) without the patients’ express consent (77.2 %).

In countries such as Germany, some hospitals have facilitated the use of CHs to final-year students [35]. Similarly, in the United Kingdom, the team in charge of attending the patient, including the students, can access the CH without the patient’s express consent [34, 36]. In the United States, this action has been permitted for years [37], and it increased to 96 % of the centres in 2016. Guimarães et al. [38] made various proposals for encouraging the use of CHs among students in Portugal. In countries in which standard access is authorized, it is generally considered an advantageous tool for the learning process [39, 40]. Having this access permits long-term follow-up of patients from diagnosis up to treatment, even once the direct relationship is over. However, this post-control has caused some ethical reactions related to the duty of training students in the right to privacy and autonomy of patients [41–43]. Students must access the CH to develop their abilities in CH use and maintenance and to understand the nuances of the EMR itself during a medical consultation [44]. Moreover, the handling of the CH is analogous to learning based on clinical cases, so that, in addition to promoting good professional conduct, it permits more active participation by the student in their training by directly applying theoretical knowledge to real cases [45–47]. This approach translates into the doctor’s social duty (distributive justice) of caring for and obtaining maximum benefits (beneficence) for all the patients. The duty to protect the patient and, at another level, to protect the medical profession is based on trust and requires honesty, integrity and dignity [48]. Therefore, although the above advantages are numerous and obvious, other authors have stated that this type of action may be potentially harmful for the patient [48]. Illegal disclosures and unethical conduct have been described as potential dangers of student misuse of CH access in relation to patient privacy [49]. Observing the principle of nonmaleficence implies that the interest of the patient should be prioritized in the absence of a clear, additional benefit to the educational objectives of the student [50].

Restricting complete access to the entire evolution of the patient and assigning levels according to the year of the student’s training [34] have been proposed for establishing some limits guaranteeing the educational objective. However, these premises should, in turn, safeguard the patient’s autonomy [45, 51]. Therefore, rather than directly constraining access, the ultimate solution to the problem could be the same as in the countries where it is permitted, i.e., to request the patient’s consent.

One notable aspect in this study as previously described is that students accessed the patient’s CH by employing the authentication mechanism of a health professional [52]. This aspect clearly emphasizes the vulnerability of the health system, which is becoming increasingly complex and fragmented, and in which the quality and safety in attending patients have become the principal foci of attention [53]. Spanish legislation does not even contemplate student access to these records, so it does not propose solutions to these problematic types of situations. In the United Kingdom, a similar phenomenon was described in primary medical care attention, so there was a proposal to assign a unique digital identity to each student that leaves an indelible and identifiable mark and is therefore susceptible to being traced [34]. To counteract the above-described conduct, it is a priority for healthcare institutions to apply educational and even motivational measures [54] to take responsibility for the risks and ethical and legal problems arising from their employment by students [52, 55, 56]. Specifically, simulation experiences and “train the trainer” models, both for students and professionals, are considered effective methods of managing ethical challenges through education and the dissemination of evidence-based EMR strategies [57].

The law is somewhat more flexible with regard to the records kept on paper. The present data reveal the high frequency with which most of the students (71.9 %) could reach the CHs without the personal and clinical data of the patients being previously dissociated, something that is contrary to pre-established norms. The aim is to preserve anonymity unless the patients themselves have expressly consented to data access, which would seem to be the definitive solution so that the student’s training is not undermined. If the use of this information has a teaching objective, the anonymization of the CH is also mandatory, although only 49.3 % alleged that they received anonymized clinical data from patients to perform their FYP [8].

Another controversial aspect is that students have information about patients outside the healthcare centre. It is a problem that partially arises due to constant technological advances, which have favoured the storage of information and images in mobile devices such as phones, tablets, USB flash drives or laptops among the students themselves [58]. This practice poses a challenge to professionalism [59] and involves concerns related to both the privacy of the patient and consent to the availability of such data [60]. Although this paper does not explore the aforementioned issue, it is highly recommended to take strong measures to protect personal data when using such devices, expressly restricting the sharing of information through social media platforms [61], health information systems [62] or computer programs that are not subject to the security systems of healthcare centres [8, 63]. However, with respect to the data on paper, this work did show that approximately 43 % of the students disposed of copies of non-anonymized patient CH reports outside the healthcare sphere, in most cases without obtaining the patients’ consent (82.1 %). The frequency of this phenomenon was not consistent with the fact that a very high percentage (90.6 %) of them were aware of the obligation of confidentiality that protected those data. Although the copies were probably supplied by the doctor in charge of the patient, the students accessed that material outside the healthcare institution. The fact that they took part in these scenarios could be the cause of the students having a greater tendency to consider certain unprofessional acts as being acceptable behaviour after their practical work [64]. Situations in which patient privacy and confidentiality are compromised have been described as a frequent event among healthcare professionals. Consequently, several studies have noted the importance of implementing continuing medical education based on greater care and management of clinical information [65, 66]. Hence, the importance of the hidden curriculum throughout pre-degree training is clear so that the students incorporate modes of behaviour taken from those of their professors/tutors beyond the contents of the formal curriculum.

This study has several limitations. The response rate was suboptimal, probably due to the dispersion of the population that belong to different universities. The voluntary nature of the survey and the paucity of institutional promotion at the faculties played havoc with participation. Another limitation of the study was that respondents were students who were aware of the issues raised by the survey and may have chosen socially accepted answers. In general terms, these limitations may have led to an underestimation of non-compliance with the *Protocol*.

## Conclusions

The findings of this study have a number of practical implications (Table 3). A large majority of the medical students were aware that the obligation of confidentiality also affected the contents of the CH (90.6 %). However, they frequently had access to CHs that were not anonymized, and almost half (43.3 %) possessed copies of non-anonymized patient CH reports outside the healthcare environment.

**Table 3:** Key points and recommendations

• The Spanish *Patient Privacy Protocol* promotes respect for patient confidentiality among all Health Sciences students.	
• Medical students were aware of the obligation of confidentiality although a high percentage possessed copies of non-anonymized patient CH reports outside the healthcare environment and were not always adequately identified.	
• Despite restrictions on access to patients' CHs, more than half of the students used a health professional's password without the prior express consent of patients and mostly without the anonymization of the CHs.	
• The role of university centres together with healthcare institutions is to promote theoretical training related to confidentiality and guarantee continuing medical education.	
• A more feasible approach would be recommendable to ensure the educational objective of having access to CHs by establishing a direct protocolized request to ask for the patient’s consent.	

Over half of the participants accessed the CHs of patients on some occasions by using the password of a health professional but 77.2 % of them had not obtained the patients’ express consent.

The role of healthcare institutions together with university centres is fundamental to ensure control procedures for the clinical documentation, as well as to promote theoretical training based on a detailed analysis of the *Privacy Protocol* by means of the study of medical ethics.

## Supplementary information

**Additional file 1.**

**Additional file 2.**

## Data Availability

The datasets used and/or analyzed during the current study are available from the corresponding author on reasonable request after approval from all the authors.

## Abbreviations

CH: Clinical history
EMR: Electronic medical record
FYP: Final year project
Q: Questionnaires
