# Supplementary material for: The role of undergraduate medical students training in respect for patient confidentiality
Source: BMC Med Educ. 2021 May 12;21:273. doi: 10.1186/s12909-021-02689-6 (PMC8117324; doi:10.1186/s12909-021-02689-6)
Supplement: Supplementary file 1 — Additional file 1. [file 12909_2021_2689_MOESM1_ESM.pdf]

Questionnaire on the right to privacy of patients during the clinical practice sessions of medical students in Spain

The aim of this questionnaire is to analyze some aspects related to the privacy of patients who are being attended in the presence of students of subjects related to Health Sciences in hospitals and health centres corresponding to their training.

This survey **is directed at degree students in the 6th year of Medicine** and is based on their experience during clinical practice sessions carried out this year. It is completely anonymous and confidential. Please answer the questions with a maximum of honesty, marking the options best suited in accordance with your experience. **Sixth year students are understood to be those** whose enrolment credits are for 70% of the subjects of the 6th year in the study plan. The time to answer the questionnaire will be not more than 5 minutes.

Age (in years)**:** *

Sex: *

- □ Female
- □ Male

In the academic year 2019/20 you have done clinical practice sessions at the .....: *

- □ Universidad Alfonso X el Sabio
- □ Universidad Autónoma de Madrid
- □ Universidad Católica de Murcia
- □ Universidad Católica de Valencia
- □ Universidad CEU Cardenal Herrera
- □ Universidad CEU San Pablo
- □ Universidad Complutense de Madrid
- □ Universidad de Alcalá
- □ Universidad de Cádiz
- □ Universidad de Cantabria
- □ Universidad de Castilla La Mancha, Campus de Albacete
- □ Universidad de Castilla La Mancha, Campus de Ciudad Real
- □ Universidad de Córdoba
- □ Universidad de Extremadura
- □ Universidad de Granada
- □ Universidad de la Laguna
- □ Universidad de las Palmas de Gran Canaria
- □ Universidad de Málaga
- □ Universidad de Murcia
- □ Universidad de Navarra
- □ Universidad de Oviedo
- □ Universidad de Salamanca
- □ Universidad de Santiago de Compostela
- □ Universidad de Sevilla
- □ Universidad de Valladolid
- □ Universidad de Zaragoza
- □ Universidad del País Vasco/ Euskal Herriko Unibertsitatea
- □ Universidad Europea de Madrid
- □ Universidad Francisco de Vitoria
- □ Universidad Internacional de Catalunya
- □ Universidad Miguel Hernández
- □ Universidad Rey Juan Carlos
- □ Universitat Autònoma de Barcelona
- □ Universitat de Barcelona, Campus de Bellvitge
- □ Universitat de Barcelona, Campus del Clínic
- □ Universitat de Girona
- □ Universitat de Lleida
- □ Universitat de València
- □ Universitat Jaume I
- □ Universitat Pompeu Fabra
- □ Universitat Rovira i Virgili

1. **The right to privacy of a patient refers to: (tick the options that you think are correct):** *
   - ◻ The confidential nature of their health data
   - ◻ Only authorized persons being able to access the above data
   - ◻ Iinformation that the patient has told us in confidence
   - ◻ None of the above

2. **The obligation of confidentiality with respect to a patient affects: (tick the options that you think are correct):** *
   - ◻ Their private health data (including degrees of disability and genetic data)
   - ◻ Data from their biographies and their environment that if known by others could affect them
   - ◻ The data contained in their clinical history
   - ◻ Data obtained by verbal communication, recordings, videos, etc.
   - ◻ An obligation that is kept up even when the patient has died
   - ◻ None of the above

3. **During the clinical practice sessions in the past year have you signed a commitment to confidentiality in order to carry them out?:** *
   - ◻ Yes
   - ◻ No
   - ◻ Don't know/ No opinion

4. **During your clinical practice sessions in the past year indicate how often you knew who was the instructor (clinical tutor) in charge of supervising your training during those sessions:** *
   - ◻ Always
   - ◻ Often
   - ◻ Sometimes
   - ◻ Seldom
   - ◻ Never

5. **During your clinical practice sessions in the past year, tick how often you wore an identification tag with your photograph, name and surname, and which was visible...:** *
   - ◻ Always
   - ◻ Often
   - ◻ Sometimes
   - ◻ Seldom
   - ◻ Never

6. **Did the fact of not wearing an identification tag result in any reaction or call to attention from your clinical session tutor?..** *
   - ◻ Yes
   - ◻ No
   - ◻ Don't know/No opinion

7. **With respect to your clinical practice sessions in the past year, tick how often you think that the patient had express knowledge of your being a degree student (instead of a resident or associate doctor, for instance)?:** *

- □ Always
- □ Often
- □ Sometimes
- □ Seldom
- □ Never

8. **With respect to your clinical practice sessions during the past year, tick how often you remember the presence of 4 or more Health Sciences students attending to one same patient:**
   - □ Always
   - □ Often
   - □ Sometimes
   - □ Seldom
   - □ Never

9. **During your clinical practice sessions in the past year, have you at any time accessed the electronic medical record of a patient using the authentification mechanism (code or password) of any healthcare professional which the latter gave to you?:**
   - □ Yes
   - □ No

10. **Did you have the express consent (spoken or written) of the patient?:**
    - □ Yes
    - □ No
    - □ Don’t know/ No opinion

11. **With respect to your clinical practice sessions in the past year, click how often you have accessed clinical histories in which the clinical data of a patient were found to be dissociated (separate) from their corresponding personal data (name, surnames, ID, etc.):**
    - □ Always
    - □ Often
    - □ Sometimes
    - □ Seldom
    - □ Never

12. **During your clinical practice sessions in the past year, have you possessed photocopies with information from the clinical history of a patient, with their respective clinical and personal data, outside the healthcare institutions where you received those sessions?:**
    - □ Yes
    - □ No

13. **Did you obtain the express consent (spoken or written) of the patient?:**
    - □ Yes
    - □ No
    - □ Don’t know/ No answer

14. **Answer this question if drafting your final degree project implies the use of the clinical data of patients: have you been supplied with patients' clinical data separated from their corresponding personal data (anonymized)?:**
   - □ Yes
   - □ No
   - □ Don't know/ No opinion

Many thanks for taking part and be kind enough to circulate this questionnaire.
